# Does thyroxine suppression therapy help to rationalize surgery in benign euthyroid nodules?

**DOI:** 10.4103/0972-3919.72688

**Published:** 2010

**Authors:** Sujata Mitra, Mukesh Jha, KM Gandhi

**Affiliations:** Department of Nuclear Medicine, Tata Main Hospital, Jamshedpur - 831 001, India; 1 Department of Surgery, Tata Main Hospital, Jamshedpur - 831 001, India

**Keywords:** Benign, diagnosis, thyroid nodule, thyroxine suppression

## Abstract

**Background:**

Nodular thyroid disease is a common endocrine problem. Most thyroid nodules are benign hyperplastic lesions, but 5–20% may be a true neoplasm. It is important to differentiate a benign from a malignant nodule early as the approach to treatment in the two is radically different. Early institution of medical management in a benign nodule may obviate the need for surgery.

**Purpose of the Study:**

The present work aims to study the efficacy of thyroxine suppression in the management of benign thyroid nodules.

**Materials and Methods:**

A prospective study on patients presenting with thyroid nodule was undertaken. The diagnostic work-up included a clinical evaluation, thyroid function tests, thyroid scintigraphy and fine needle aspiration cytology. Based on the investigations, patients were segregated in Group A (toxic nodular goiter), Group B (benign euthyroid nodule) and Group C (malignant nodule). Group A patients were managed with antithyroid drugs and radioiodine and Group C patients were managed surgically. Group B patients were put on thyroxine suppression. Patients who failed to show reduction in size of the nodule at 18 months were treated surgically.

**Conclusion:**

The response rate of benign euthyroid nodule to thyroxine suppression was 76% in the present study.

## INTRODUCTION

Nodular thyroid disease is one of the commoner endocrine problems. Most thyroid nodules are benign hyperplastic lesion but 5 to 20% of thyroid nodules are true neoplasm.[1] The prevalence of thyroid nodules varies considerably depending on a variety of factors that include iodine intake within a given population, age, sex, diet, therapeutic and environmental radiation exposure. There is age related increase in nodularity and volume of thyroid gland. Small thyroid nodules are commonly found in patients with concurrent history of Hashimoto’s Thyroiditis.[2]

The importance of managing a thyroid nodule is in the early differentiation between benign and malignant nodules, since the approach to treatment in the two is radically different. Also, medical management in a benign nodule may obviate the need for surgery and morbidity associated with it. Thyroxine suppression therapy is in use in the medical management of euthyroid benign nodules.[3–5]

However, the AACE does not include Thyroxine Suppression in its recommendation for management of benign euthyroid nodule.[6]

The aim of the present study is to evaluate the efficacy of thyroxine suppression therapy in benign euthyroid nodule, which would result in rationalization of surgery in such patients.

## MATERIALS AND METHODS

This prospective study was carried out in an 890-bedded secondary care industrial hospital in Eastern India, at the Thyroid Clinic, Department of Nuclear Medicine, a referral center for thyroid disorders.

The study group included all patients presenting with a thyroid nodule in the Thyroid Clinic between April 2004 and March 2009.

A detailed clinical examination was performed in all cases followed by biochemical tests, fine needle aspiration cytology (FNAC) of the nodule and thyroid scintigraphy.

### Clinical evaluation

The clinical evaluation included history taking, record of presenting symptoms with their duration, a systemic examination and local examination of the thyroid swelling and the neck [Table 1].

**Table 1:** Clinical evaluation

Area	Details covered
Patient details	Age, sex
Presenting symptoms and past history	Duration, type of symptom, menstrual disorders, family history, drug history
General physical examination	Weight, BP, pulse rate, pallor, respiratory rate, pedal edema, lymphadenopathy, myopathy, ophthalmopathy
Local examination of the neck	Goiter: grade, type (diffuse or nodular), mobility, bruit Cervical lymphadenopathy

### Biochemical examination

#### Serum total triiodothyronine and serum total thyroxine estimation

The quantitative measurement of serum Serum total triiodothyronine (T_3_) and Serum total thyroxine (T_4_) was carried out by radioimmunoassay (RIA) using RIA kits supplied by the Board of Radiation and Immunotechnology (BRIT), Mumbai, India (normal range of T_3_: 0.7-2.0 ηg/ml, T_4_: 5.5-13.5 μg/ml).

#### Serum thyroid stimulating hormone (TSH) estimation

Serum TSH was measured by the immunoradiometric assay (IRMA) technique using BRIT kits (normal range 0.3–6.5 μIU/ml).

### FNAC

Patients were referred to the Department of Pathology, Tata Main Hospital, for FNAC of the nodule. The FNAC findings were reported as thyroid cyst (clumps of benign follicular cells with colloid background, reduction in size after aspiration), colloid goiter with or without cystic degeneration (sheets of uniform follicular cells with a colloid-mixed hemorrhagic background) or thyroid neoplasm (moderate to highly cellular aspirate, scanty colloid, presence of lymphocytes). The indeterminate FNAC reports were those with no specific findings diagnostic of either benign or malignant pathology.

### Thyroid scintigraphy

A Technetium-99m sodium pertechnetate (Tc-99m pertechnetate) thyroid scan was performed on a GE Milleneum Dual Head Gamma Camera. manufactured by GE Medical System Israel, Tirat Hacarmel, Israel, Model No: H 3000ZL. Static image in the anterior view was taken 20 min following Tc-99m pertechnetate injection at a dose of 5.0–6.0 mCi. The total thyroid pertechnetate uptake was calculated. Presence of focal abnormality-absence of uptake (“cold” area) or increased uptake (“hot area”) was noted.

### Exclusion criteria

Based on the initial clinical and biochemical evaluation and thyroid scan, the following patients were excluded from the study:

1. Goitrous hypothyroidism
2. Physiological goiter (prepubertal and pubertal)
3. Diffuse euthyroid goiter

The study group was divided into three groups: A, B and C.

#### Group A

Patients with toxic goiter, which included autonomous functioning thyroid nodule (AFTN) and toxic multinodular goiter (TMNG), were diagnosed by thyroid function tests and thyroid perchnetate scan and uptake.

#### Group B

Patients with a provisional diagnosis of benign euthyroid nodule were diagnosed on the basis of thyroid function tests and FNAC.

#### Group C

Patients with thyroid malignancy. The provisional diagnosis of thyroid neoplasm was based on clinical, scintigraphic and FNAC findings.

The management protocol followed in the three groups was as follows:

#### Group A (AFTN and TMNG)

All patients were put on Carbimazole therapy. The loading dose was 30 mg daily in divided doses, which was tapered to a maintenance dose of 15 mg daily after 3 months. The thyroid function test was repeated after 3 months. All patients received adjuvant therapy with β-blockers. Patients that relapsed after 18 months of antithyroid drugs were treated with radioiodine ablation.

#### Group B (benign euthyroid nodule)

Patients in Group B were put on thyroxine suppression at a dose of 50–100 μg daily. TSH was kept in the low normal range for a period of 6–18 months. Patients were followed-up monthly to note the size of the nodule by clinical palpation and also to monitor the possible iatrogenic hyperthyroidism. TSH was repeated once every 6 months. Surgery was carried out in cases where the nodule did not regress after 18 months of thyroxine suppression.

#### Group C (thyroid malignancy)

Patients were subjected to definitive surgery followed by radioablation.

Patients with a strong clinical suspicion of malignancy or those with indeterminate cytology on FNAC were subjected to thyroid biopsy.

### Observations

The study group consisted of 52 patients presenting with thyroid nodule.

#### Group A (AFTN and TMN goiter)

**Figure 1:**
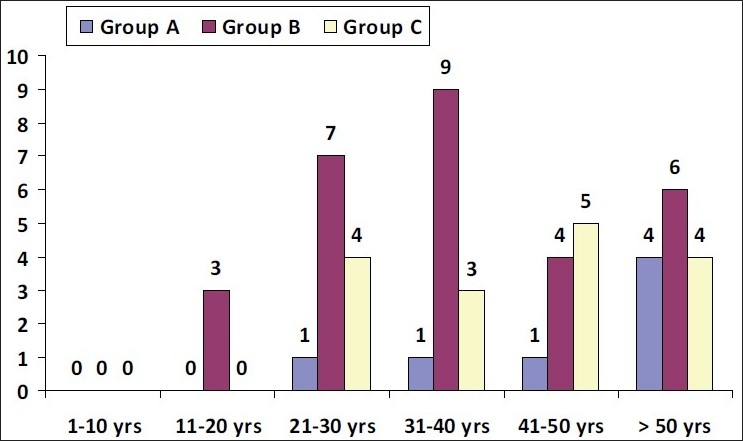
Age distribution

This group consisted of seven patients. The male to female ratio was 2:5. The mean age was 51.3 years. There were no patients below 20 years; three (43%) patients were between 21 and 50 years and four (57%) patients were above 50 years of age [Table 2a and Figure 1]. The disease duration was <3 years in two (28%) of the seven patients. Weight loss and insomnia were the most common symptoms. One patient had thyroid ophthalmopathy. In all seven patients, the thyroid technetium scan showed a raised total uptake, ranging from 10.4% to 24.0% (normal range of uptake = 0.4-4.0%).

**Table 2a:** Age distribution

Age (years)	Number of cases		
	Group A (n = 7) (toxic goiter)	Group B (n = 29) (benign euthyroid nodule)	Group C (n = 16) (malignant nodule)
1–10	-	-	-
11–20	-	3	-
21–30	1	7	4
31–40	1	9	3
41–50	1	4	5
>50	4	6	4

Three (43%) patients continued to be toxic after 18 months of Carbimazole therapy. Two of these received radioiodine ablation and one underwent subtotal thyroidectomy. One patient relapsed postradioiodine therapy and subsequently underwent subtotal thyroidectomy.

#### Group B (provisionally diagnosed as benign euthyroid nodule)

This group had 29 patients and the male:female ratio was 3:26 [Table 2b]. The mean patient age was 39.6 years. There were three patients below 20 years. Twenty (70%) patients were between 21 and 50 years and six patients were above 50 years of age. All three male patients were above 40 years of age, whereas females were present in all age groups, from 11 years to above 50 years.

**Table 2b:** Sex distribution

Sex	Group A (n = 7)	Group B (n = 29)	Group C (n = 16)
Male	2	3	2
Female	5	26	14

**Figure 2:**
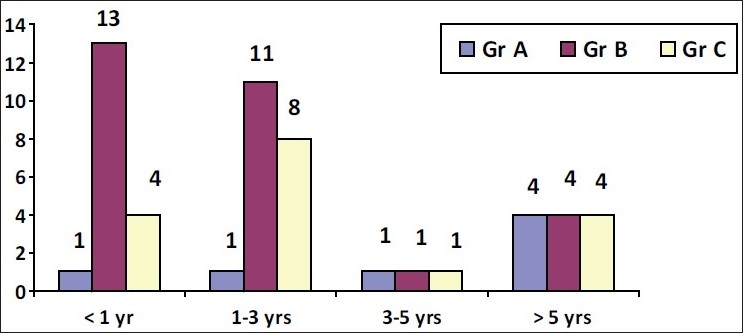
Duration of nodule

In 13 of the 29 patients (45%), the duration of nodule was <1 year [Table 3 and Figure 2].

**Table 3:** Duration of nodule

Years	Number of cases		
	Group A (n = 7)	Group B (n = 29)	Group C (n = 16)
<1	1	13	4
1–3	1	11	8
3–5	1	1	1
>5	4	4	4

The FNAC finding in the 29 patients was as follows: colloid goiter – 24, colloid goiter with cystic degeneration – 1 and thyroid cyst – 4 [Table 4].

**Table 4:** FNAC findings in Group B

Cytological findings	Number of cases
Colloid goiter	25
Thyroid cyst	04

The thyroid pertechnetate total uptake was in the normal range in all 29 cases. Ten of the 29 patients showed presence of a “cold” area in the scan. In all these 10 patients, the FNAC finding was that of colloid goiter, but the clinical suspicion of malignancy was high in seven of them. Thyroid biopsy was performed in these seven patients with a clinical suspicion of malignancy, FNAC finding of colloid goiter and “cold” area on technetium scan. The histopathology revealed thyroid neoplasm in all seven cases, and these were managed surgically, together with the Group C patients.

Thyroxine suppression at a dose of 50–100 μg daily was given in 22 of the 29 Group B patients, seven having undergone biopsy and subsequent surgery. These included four cases of thyroid cysts, which were aspirated and subsequently put on thyroxine suppression. A reduction in the size of the nodule following thyroxine suppression was seen in 16 (66%) patients, with complete disappearance of nodule seen in six patients at 12 months follow-up [Table 5]. In three patients, no reduction in size of nodule was seen and in another two, it gradually enlarged despite thyroxine suppression. In one patient with a thyroid cyst, there was recurrence in spite of repeated aspiration and thyroxine suppression. Therefore, six of the 22 patients did not respond to thyroxine suppression and had to be subjected to surgery.

**Table 5:** Nonresponders to thyroxine suppression in Group B

Category	Regression after thyroxine	No response to thyroxine
Group B (benign euthyroid nodule)	27	6

#### Group C (malignant nodule)

**Figure 3:**
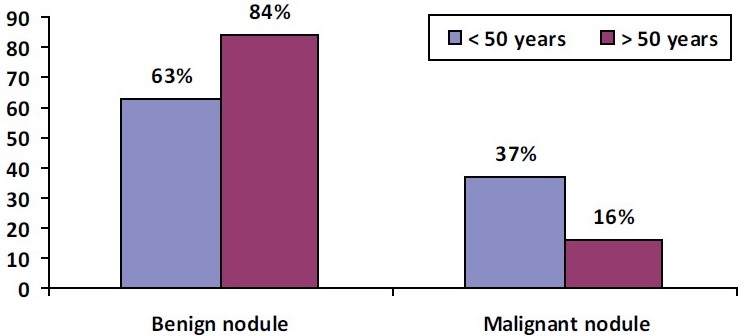
Distribution of benign and malignant nodule by age

This group consisted of 16 patients. The male:female ratio was 2:14. The mean age was 43.6 years and the mean duration of disease was 22.4 months. There were three patients above 50 years, while the remaining were between 21 and 50 years [Table 6] [Figure 3].

**Table 6:** Distribution of benign and malignant nodule by age

Age	Benign nodule	Malignant nodule
<50 years	12	7
>50 years	21	3 + 1 inconclusive

Besides the thyroid nodule seen in all 16 patients, six patients presented with lymphadenopathy and one patient presented with dysphagia.

The FNAC in the 16 cases was reported as follows: follicular neoplasm in 15, inconclusive in one patient [Table 7].

**Table 7:** FNAC findings in Group C

Cytological findings	Number of cases
Follicular neoplasm	15
Inconclusive	1

The technetium thyroid scan showed a “cold” area of absent tracer uptake in 12 patients. In four patients, this was reported as normal.

Total thyroidectomy followed by radioablation was performed in 10 of the 16 cases. In six cases, subtotal thyoidectomy was carried out.

Subtotal thyroidectomy was also performed in two of the Group A patients who relapsed after radioiodine therapy and in six of the Group B patients where there was failure of thyroxine suppression. Another seven patients of Group B who underwent biopsy, based on a high clinical suspicion even though the FNAC was negative for malignancy, were also subjected to surgery, and three of these patients were diagnosed as follicular carcinoma. Therefore, of a total of 52 patients, ultimately 31 underwent surgery, and 19 of these were thyroid malignancy [Tables 8 and 9].

**Table 8:** Reasons for surgery

Reason for surgery	Number of cases
FNAC – follicular neoplasm	15
FNAC – indeterminate	01
FNAC – colloid goiter; thyroid scan – “cold” area; clinically malignant	07
Relapse after antithyroid drugs in toxic nodule (large goiter – 01, relapse after radioiodine – 01)	02
Failure of thyroxine suppression in benign euthyroid nodule and thyroid cyst	06

**Table 9:** Type of surgery

Type of surgery	No. of patients
Hemithyroidectomy/lobectomy	12
Subtotal thyroidectomy	5
Total thyroidectomy	10

## DISCUSSION

In a review article, Mazzaferri[7] reports an increase in prevalence of thyroid nodules with age and a prevalence of 5% in patients at 50 years found by manual palpation. The ultrasonography (USG)-detected prevalence is far higher, almost 55%. Less than 1% of the nodules are reported to cause hyperthyroidism.[8] In surgically removed thyroid nodules, Christensen has reported malignancy in 18 of 100 surgically removed thyroids.[9] FNAC has a sensitivity of 65–99% and a specificity of 72–100% in diagnosing a thyroid malignancy. It is the most effective method in detecting benign and malignant thyroid lesion.[10]

Thyroid hormone therapy for nodular goiter, for cosmetic reasons or to avoid diagnostic and therapeutic surgery, results in moderate to complete regression of the nodule.[3] Thyroxine suppressive therapy is an appropriate alternative as long as the patient is followed-up carefully at 6-month intervals.[11] It is prescribed in doses sufficient to suppress the TSH level to 0.1–0.5 μU/ml for 6–12 months; more prolonged therapy is reserved for patients in whom decrease in nodule size is documented on USG. After 12 months, the dose of thyroxine is decreased to maintain the serum concentration of TSH in the low-normal range. The AACE/AME task force guidelines[6] regarding thyroxine suppressive therapy for FNAC-negative thyroid nodules restricts its use to patients from iodine-deficient areas, young patients with small goiter and nodules with no evidence of functional autonomy (Grade C level of evidence). It further states that thyroxine suppression should be avoided in large goiter, clinically suspicious lesion or those with inadequate cytologic sampling, in postmenopausal women or patients with osteoporosis and cardiovascular disease. Reports also suggest that thyroxine suppression for benign nodule does not conclusively result in shrinkage of nodules, although a short trial of suppression may be reasonable in selected patients.[12] A study by La Rosa[13] has shown reduction in size of the colloid and degenerative nodules but not in hyperplastic or fibrotic nodules.

In the present series (*n* = 52), most patients presented in their 3^rd^ and 4^th^ decade, with 73% of the patients below 50 years. The duration of the thyroid nodule was 1–3 years in 73.2% of the cases, between 3–5 years in 7.7% and >5 years in 27.7%. Among the three groups, Group B (benign euthyroid nodule) patients presented at an earlier age, with the mean age being 39.6 years compared with 51.4 years in Group A (TMN goiter) and 43.6 years in Group C (malignant nodule). The disease duration was also lower in Group B patients, 17.8 months compared with 54.7 months in Group A and 22.4 months in Group C.

The present study did not include USG-detected thyroid nodules detected in an otherwise asymptomatic patient.

The incidence of toxic nodule in the present series was 16%, higher than that reported in other series.[8] Nineteen of the 52 patients in our series were diagnosed as thyroid malignancy.

Ten of the 44 euthyroid nodules were reported malignant on FNAC. It was false-negative in six cases and indeterminate in one case.

Twenty-two patients with euthyroid benign nodule were put on thyroxine suppression. A reduction in the size of the nodule following thyroxine suppression was seen in 76% of the cases, with complete disappearance of nodule seen in six patients (24%) at 12 months follow-up. In three patients, no reduction in size of the nodule was seen and in two, it gradually enlarged despite thyroxine suppression at 18 months. In one patient with a thyroid cyst, there was recurrence in spite of repeated aspiration and thyroxine suppression.

Thirty-one of 52 patients underwent surgery. The most common indication of surgery was malignancy in 19 patients (36%). Seven patients were reported as colloid goiter on FNAC. However, there was a “cold” area on the thyroid scan and the clinical suspicion of malignancy was high therefore these were subjected to surgery. Five patients with a benign nodule and two patients with a toxic nodule underwent surgery after failure of medical treatment. A thyroid cyst recurred after re-aspiration, and was treated surgically.

The response rate of 76% to thyroxine suppression in the present study was higher than that in other studies, where it is reported to range from 17%[8] to 50%.[13] The reason could be a greater number of younger patients (73% presented below 50 years of age). Also, in the majority of the patients, the duration of nodule was <3 years. An earlier age of presentation and shorter duration of the disease may have contributed to a better response to thyroxine.

## CONCLUSION

An initial diagnostic work-up for thyroid nodule that included thyroid function tests and FNAC helped to establish the thyroid functional status and cytology of the nodule. This was used to differentiate between euthyroid benign and malignant nodules. Thyroid scan was used only to establish the functional status of the thyroid tissue. FNAC remained the mainstay of diagnosis, helping to diagnose malignant nodules, with the thyroid pertechnetate isotope scan providing corroborative evidence. However, a clinical correlation and thyroid scan were used to decide management in patients that were FNAC negative but with a clinical suspicion of malignancy. USG was not performed and clinical follow-up was used to document disease regression or progress. This remains a drawback of the study. Also, a long-term follow-up would be required to document recurrence of nodule.

Thyroxine suppression in benign euthyroid nodule shows a good response rate if the therapy is instituted early in the disease process. The response is better in younger patients. The need for surgery can be rationalized in such cases and should be undertaken only if there is no response after a 1-year follow-up.
